# Concomitant fractures in patients with proximal femoral fractures lead to a prolonged hospital stay but not to increased complication rates or in-house mortality if treated surgically: a matched pair analysis

**DOI:** 10.1007/s40520-023-02348-4

**Published:** 2023-01-25

**Authors:** Annabel Fenwick, Michael Pfann, Jakob Mayr, Iana Antonovska, Franziska Von der Helm, Stefan Nuber, Stefan Förch, Edgar Mayr

**Affiliations:** 1 Department of Trauma, Orthopedic, Plastic and Hand Surgery, University Hospital of Augsburg, Stenglinstrasse 2, 86156 Augsburg, Germany; 2 Zentrum Für Unfallchirurgie Und Orthopädie, Klinikum Ingolstadt GmbH, Krumenauerstraße 25, 85049 Ingolstadt, Germany

**Keywords:** Complications, Concomitant fractures, Fragility fractures, Geriatric trauma, Mortality, Proximal femur fracture

## Abstract

**Background:**

Impact of concomitant fractures on patients sustaining a proximal femur fracture remains unclear. Rising numbers and patient need for rehab is an important issue. The objective of our study was to investigate the impact of concomitant fractures, including all types of fractures, when treated operatively, for proximal femur fractures on the length of hospital stay, in-house mortality and complication rate.

**Methods:**

Observational retrospective cohort single-center study including 85 of 1933 patients (4.4%) with a mean age of 80.5 years, who were operatively treated for a proximal femoral and a concomitant fracture between January 2016 and June 2020. A matched pair analysis based on age, sex, fracture type and anticoagulants was performed. Patient data, length of hospital stay, complications and mortality were evaluated.

**Results:**

The most common fractures were osteoporosis-associated fractures of the distal forearm (*n* = 34) and the proximal humerus (*n* = 36). The group of concomitant fractures showed a higher CCI than the control group (5.87 vs. 5.7 points; *p* < 0.67). Patients with a concurrent fracture had a longer hospital stay than patients with an isolated hip fracture (15.68 vs. 13.72 days; *p* < 0.056). Complications occurred more often in the group treated only for the hip fracture (11.8%, *N* = 20), whilst only 7.1% of complications were recorded for concomitant fractures (*p* < 0.084). The in-house mortality rate was 2.4% and there was no difference between patients with or without a concomitant fracture.

**Conclusions:**

A concomitant fracture to a hip fracture increases the length of hospital stay significantly but does not increase the complication rate or the in-house mortality. This might be due to the early mobilization, which is possible after early operative treatment of both fractures.

## Background

The number of proximal femur fractures is estimated to rise to more than 4.5 million by 2050 [1–4]. Not only are they linked to an exceedingly high mortality rate within the first postoperative year, they also reduce patient mobility and self-sustainability and lead to an impairment of most daily life activities [5–7]. A high proportion of patients need caretaking facilities postoperatively [8]. The economic burden is already by far exceeding health care resources [3, 9]. Treatment consists of arthroplasty or osteosynthesis but depending on fracture morphology especially for femoral neck fractures arthroplasty should be considered for geriatric patients as osteosynthesis failure rate has to be taken into account [10].

Underlying diseases such as diabetes and cardiovascular disorders as well as cognitive impairment have been linked by studies to a higher mortality rate for patients suffering from hip fractures [11, 12]. Women and patients with osteoporosis are at even higher risk sustaining a hip fracture [13]. This leads to a significant number of patients presenting to A&E with concomitant, often osteoporosis-associated fractures. The prognostic value of these concomitant fractures remains unclear, and data are limited [14, 15]. Literature has discussed concomitant fractures as disadvantageous as they lead to lower functionality and higher mortality [8, 16–19]. But the data are mainly limited to concomitant fractures of upper limbs and leaves the specific treatment of the additional fractures open. If there is a disadvantage, the importance of identifying these patients lies in the opportunity to enable more intensive rehabilitation and increase early mobility for a possible return to daily activities and independency.

The objective of our study was to investigate the impact of concomitant fractures, including all types of fractures, when treated operatively, for patients with proximal femur fractures with regards to the length of hospital stay, in-house mortality and complication rate.

## Material and methods

### Data acquisition

For our retrospective cohort single centre study (Level III) all patients treated operatively for a proximal femoral fracture (femoral neck, pertrochanteric and subtrochanteric fractures) at our level I trauma centre between January 2016 and June 2020 were evaluated. Exclusion criteria were: primary conservative treatment, greater trochanteric fractures, periprosthetic fractures as well as referrals for revision surgery and polytraumatised patients.

The study conducted was approved by the local Ethics Committee and fulfils the standards of the declaration of Helsinki (20-2155-101).

1933 patients were treated for proximal femur fractures in the mentioned period. Of these patients 95 (4.91%) presented with a concomitant facture at the time of admission. 85 patients were enrolled in our study group as the second fracture was treated surgically during the same hospital admission.

### Matched pair analysis

Patients with concomitant fractures at the time of admission which were subsequently treated operatively during the same hospital stay were extracted to form our study group. A matched pair analysis was carried out. We formed a control group without concomitant fractures which was matched on 4 criteria: age, gender, fracture morphology and anticoagulant medication.

The charts were reviewed for demographic data such as age, gender, BMI, comorbidities including the Charlson Comorbidity Index CCI [19] and ASA American Society of Anaesthesiologists classification [20], fracture morphology, co-geriatric management, medication. Type of surgery and time to surgery from admission were evaluated.

Outcome measures were the length of stay in the intensive care unit as well as the overall length of hospital stay (LHS) and In-house mortality. Complications were analysed and divided into urinary infections, pneumonia, embolism or thrombosis, haematoma, wound infections, mechanical complications, i.e. postoperative fracture or dislocation or cutting out.

### Therapy

One consistent therapy protocol was applied throughout the total period reviewed. Target time to surgery was within 24 h of admission for all patients without anticoagulation or only anti-platelet therapy (AP), including dual AP therapy. Patients with DOACs (Edoxaban, Rivaroxaban, Apixaban) were divided into two groups according to their kidney function (Gr 1: GFR > 50, Gr 2: GFR < 50). If renal clearance was good, surgery was performed within 24 h. If renal function was impaired, surgery was postponed to 24–48 h after admission to reduce risk of bleeding. Depending on pre-operative mobility, comorbidities and fracture morphology total or hemi arthroplasty (cemented or uncemented, Fa. Zimmer Biomet Indiana, US) was performed for femoral neck fractures, intramedullary nailing PFNa, Fa. Synthes Oberdorf, Switzerland, (± cerclage) for pertrochanteric fractures and plate/screw osteosyntheses (DHS, dynamic hip screw, Fa. Synthes) for undisplaced pertrochanteric or lateral femoral neck fractures. The subtrochanteric fractures were addressed by open reduction, cerclage and cephalomedullary nailing in side-positioning. 30 min prior to surgery all patients received an i.v. single shot of 2 g Cefazolin.

Postoperatively, venous thromboembolism prophylaxis was given from day one with Enoxaparin 40 mg subcutaneously. Anticoagulants were substituted with Tinzaparin-Sodium according to patient weight postoperatively. All patients were allowed full weight bearing immediately after surgery and received physiotherapy from day one. In case of a hindfoot fracture full weight bearing was allowed with a VACOped boot.

### Statistical analysis

Statistical analysis was carried out with IBM SPSS Statistics (version 27; IBM Deutschland Ltd., Ehningen, Germany). Normal distribution of all data was verified. The student’s t-test and chi-square were used to determine influencing factors regarding complications and mortality; 95% confidence intervals and standard deviations were calculated. For data without normal distribution the Wilcoxon Rank Test was used. We used Fisher’s exact test for the description of significant differences in mortality between the groups. The significance level was set at 5% (α = 0.05).

## Results

The average age was 80.5 years (range: 34–99; SD 10.8). 74.1% were female and 25.9% male. The mean BMI was 24.35 kg/m^2^ (range: 14.8–38.1 kg/m^2^). Each group of 85 patients comprised 37 femoral neck fractures, 41 pertrochanteric and 7 subtrochanteric fractures. In 36 cases total hip endoprothesis was implanted and 33 patients received a hemiarthroplasty. Cephalomedullary nailing was done in 97 cases and osteosynthesis with dynamic hip screw in four cases. Anticoagulant therapy was recorded for 82 patients (48.2%). 56 patients had antiplatelet therapy and 26 were on either Warfarin or DOACs.

### Concomitant fractures

The 85 identified patients with concomitant fractures had 92 fractures. The most common fractures were osteoporosis-associated fractures of the distal forearm (*n* = 34) and the proximal humerus (*n* = 36) followed by fractures of the distal humerus (*n* = 4) and the olecranon (*n* = 4). The distal forearm fractures were all treated with locking plate osteosynthesis and the olecranon fractures by tension band wiring. Patients with distal humerus fractures were treated with elbow arthroplasty. Of all the patients with proximal humerus fractures 6 obtained reverse shoulder arthroplasty whilst the other patients were treated with plate- or nail osteosynthesis. Two patients each were also surgically treated for spine fractures, patella fractures, clavicle—and tibial shaft fractures. Furthermore, there was one patient with a talus fracture, one with a calcaneus fracture and one with a metatarsus V fracture. One patient was treated for a metacarpal fracture and one for a radial head fracture. In one case there was a periprosthetic proximal tibia fracture, which received revision arthroplasty.

### Preoperative status

**Fig. 1:**
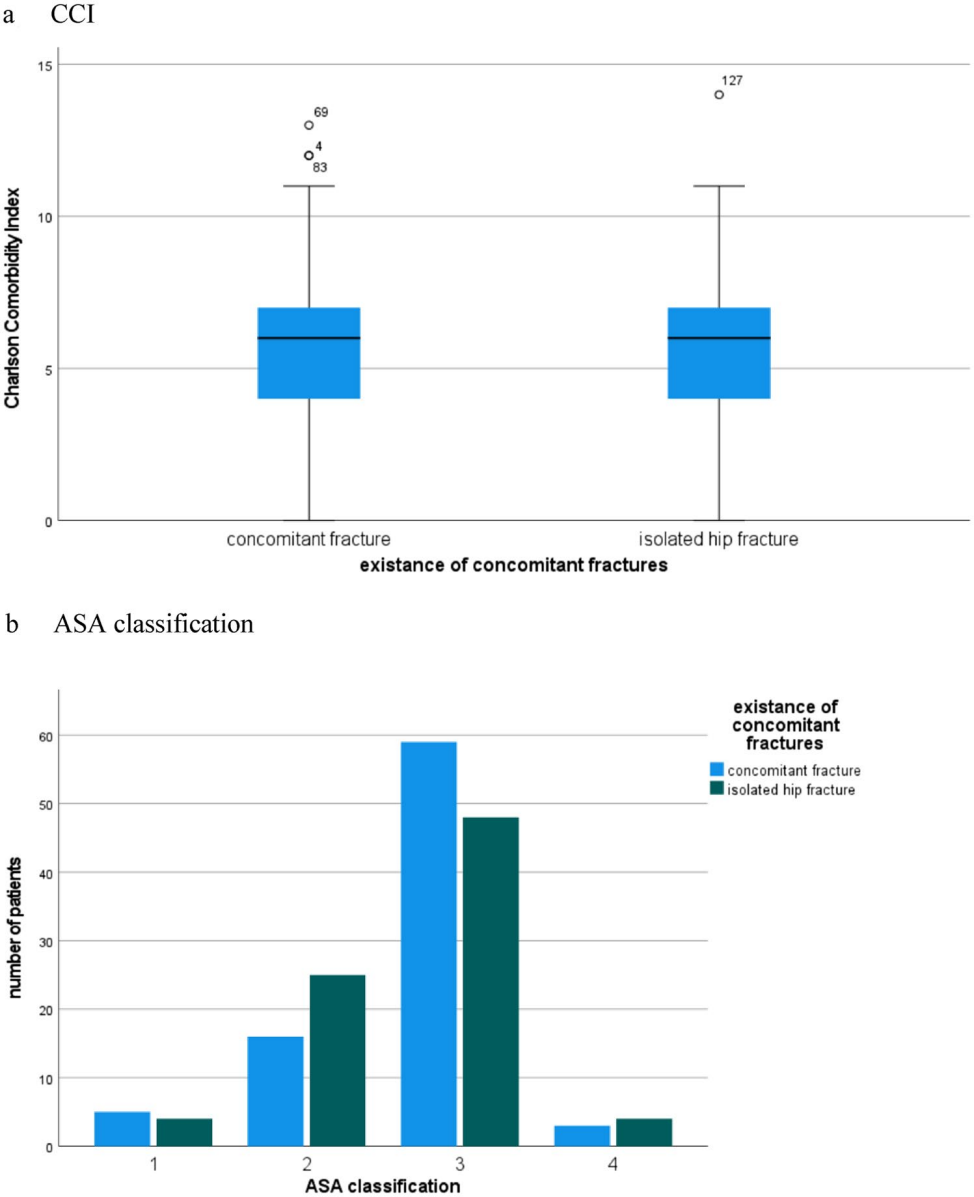
The average comorbidities **a** Charlson Comorbidity Index and **b** ASA classification between patients with concomitant fractures and isolated hip fractures. **a** CCI, **b** ASA classification

The average CCI for the total cohort was 5.79 points (range: 0–14, SD 2.5). The group of concomitant fractures showed a slightly higher but not significant CCI in comparison to the control group (5.87 vs. 5.7points; *p* < 0.67). The ASA classification was also distributed equally amongst both groups with most patients classified ASA II and III (90.2%) (Fig. 1).

110 patients (64.7%) had been self-sustaining without caretaking prior to hospital admission. The distribution of amount of caretaking was similarly distributed in both groups (level 1: 12, level 2: 20, level 3: 196; level 4: 18, level 5: 10). Preoperative mobility was assessed and already reduced in 47.6% of the cohort i.e., need of at least a cane or a walker whilst 52.4% had no restrictions in walking or distance of walking.

### Time to surgery

The time from admission to surgery for all patients was on average 24.95 h (range: 2.16–107.18; SD: 17.7). Both groups showed no significant difference in the waiting time to surgery (concomitant: 25.94 h vs. 23.95 h; *p* < 0.466).

### Length of hospital stay

**Fig. 2:**
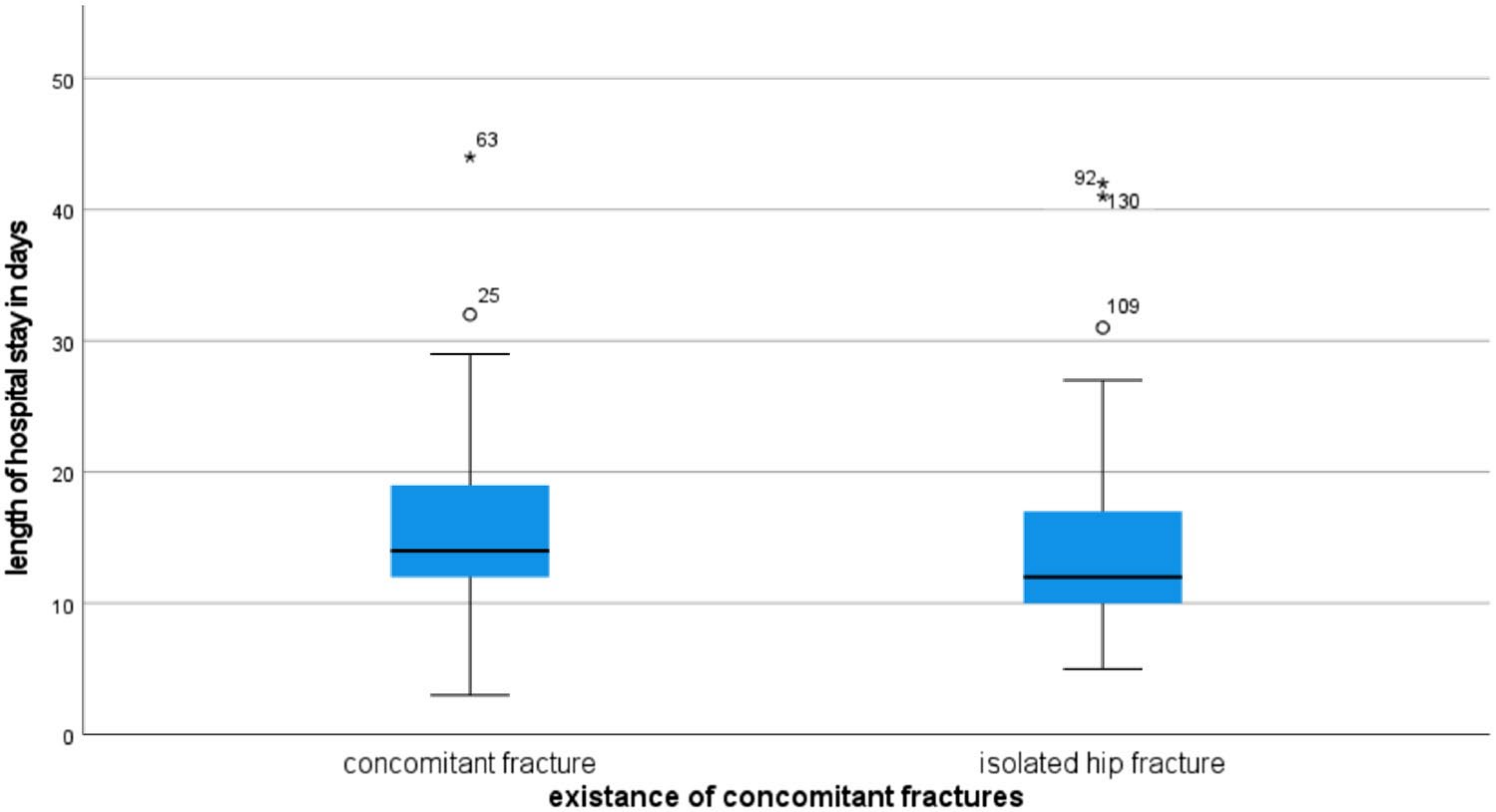
Comparison of length of hospital stay for hip fractures only and concomitant fractures

The average LHS for the entire cohort was 14.7 days (range: 3–44; SD 6.6). Patients who presented with a further treated fracture had a mean longer hospital stay than patients with an isolated hip fracture (15.68 vs. 13.72 days; *p* < 0.056) (Fig. 2). In addition, the length of stay postoperatively in the intensive care unit ICU was significantly longer for patients treated for more than one fracture (1.01 vs. 0.45 days; *p* < 0.024).

### Complications and mortality

The entire cohort showed a complication rate of 18.8%. In total complications occurred more often in the group treated for only the proximal femur fracture (11.8%, *N* = 20) whilst only 7.1% of complications were recorded for the group with concomitant fractures (*p* < 0.084). Pneumonia occurred 5 times in both groups, whilst urinary tract infections were more common in the group with isolated hip fracture (5.9 vs. 12.9%; *p* < 0.08). In each group, there was one case (1.2%) of deep wound infection with the need for surgical revision surgery. Blood loss was significantly higher in the group treated for more than one fracture (1711.09 vs. 1326.2; *p* < 0.007).

**Fig. 3:**
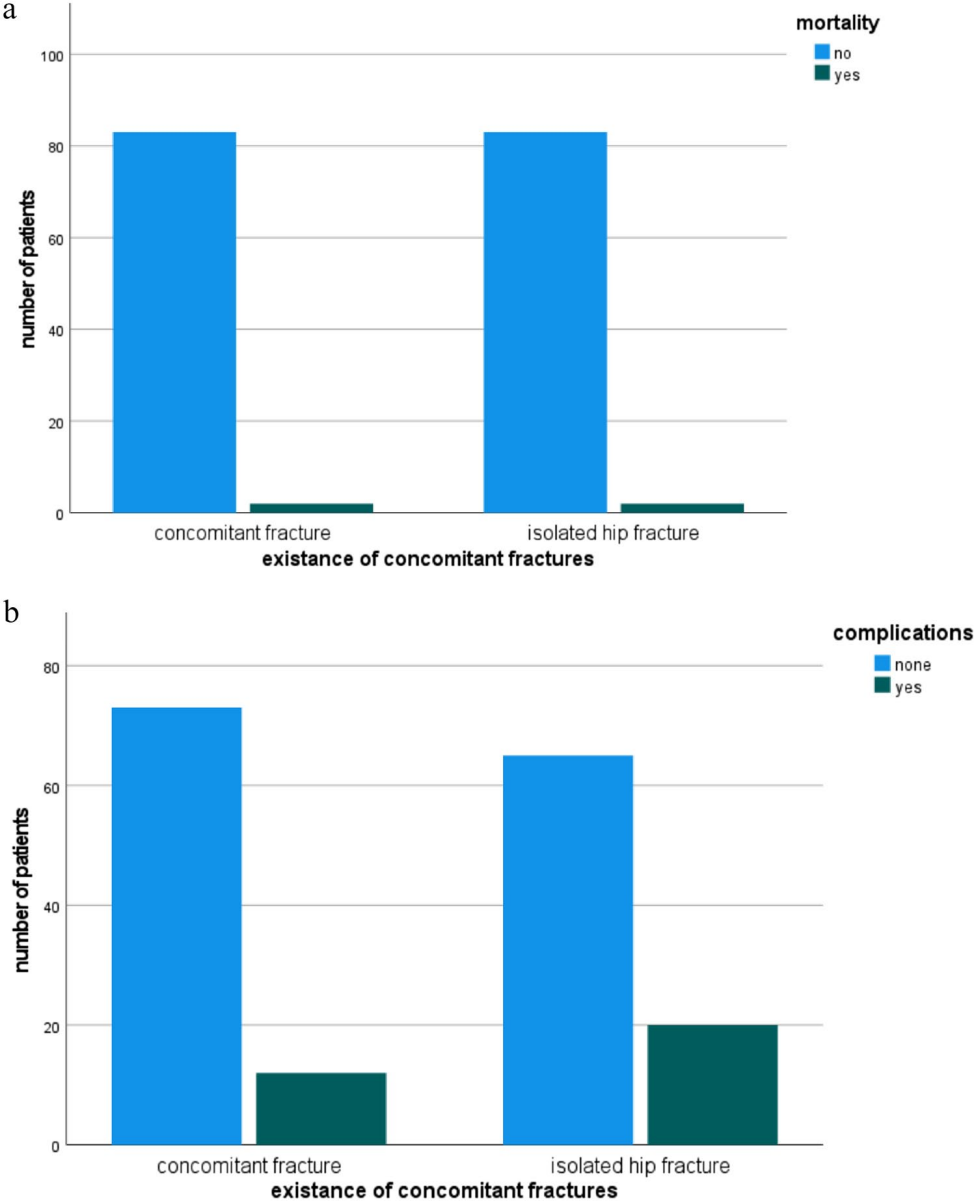
**a** Comparison of mortality rate between the two matched groups. **b** Comparison of complication rate between the two matched groups

The overall in-house mortality rate was 2.4% (*N* = 4). There were 2 patients with concomitant fractures and 2 patients with a single proximal femur fracture who died postoperatively (Fig. 3). Death causes were pulmonary embolism, cardiac arrest and pneumonia.

## Discussion

There is no question that patients suffering from proximal femur fractures have a high impact on their daily life and a high mortality risk [21]. Furthermore, geriatric patients are particularly vulnerable and often show a worse outcome. Therefore, Di Monaco [22] raised the question of whether a further coinciding fracture could actually lead to an even worse outcome or a higher mortality rate. The percentage of patients affected in our study is similar to previously published studies (3.7–6.5%) making up about 5% of the patients presenting with proximal femur fractures [17, 23, 24]. This may seem a rare condition, but the relevance of the topic is marked by a continuously rising aging population of which 5% makes a substantial number of patients in need of special treatment and rehabilitation.

Uzoigwe [23] demonstrated women are more likely to suffer from a hip fracture and a further fracture whilst Mulhall [25] concluded that higher age was associated with the occurrence of a concurrent fracture. A study conducted by Di Monaco et al. was able to prove that Geriatric Nutritional Risk Index GNRI scores were significantly lower in the subgroup of women with hip fracture and concurrent upper-extremity fracture than in the control group and they concluded that a low GNRI score may have an influence on the genesis of the concurrent fractures [26].

As most studies are restricted to upper limb fractures as the concomitant injury, we enrolled all types of fractures. In agreement with Robinson et al. [16] we also found the most common fractures to be associated with proximal femur fracture in our cohort to be fractures of the distal forearm and proximal humerus making up more than 75% of all the fractures included.

A large metanalysis on the topic by Kim et al. involving 217.233 patients with hip fractures and concurrent upper limb fractures found a higher 30-day mortality rate but no difference in the long-term mortality rate [8]. Higher mortality rates for concomitant fractures were also seen by Mulhall et al. [25] (10.3% vs. 5.6%) and Buecking et al. [17] (increase of 1.8%). Furthermore, a study conducted by Thayer et al. [18] concluded that this patient group was at a higher risk for in-house mortality than patients with an isolated proximal femur fracture. Our results agree with Ng et al. in not finding any difference in mortality rates amongst the groups compared [27]. Ng et al. found increase of age was linked to 30-day mortality. Our mortality rate for concomitant fractures at 2.4% (4% (*N* = 78) for the overall cohort of 1933 [28] seems relatively low in this cohort but may be linked to our early mobilization program with full weight bearing after surgery for both fractures to diminish complications linked to prolonged immobilization. Furthermore, our geriatric patients are treated on an orthogeriatric ward and studies have been able to show that an interdisciplinary orthogeriatric approach reduces in-house mortality and improve the functional outcome postoperatively, for example, the capability of living at home post-surgery as well as mobility.

[29]. Combined orthogeriatric treatment seems to become even more relevant if more than one injury is preexistent.

All studies agree on the fact that concomitant fractures lead to longer hospitalization [18, 23, 30, 31]. Di Monaco [22] further evaluated the types of fractures and found a prolonged length of stay for patients with a proximal humerus fracture but not for fractures concerning the distal forearm whilst Kim et al. [8] did not differentiate between the types of upper limb fractures and saw an increase in length of hospital stay for mean 1.67 days which is very similar to the results of our cohort showing an increase of the average 1.96 days. The largest single study conducted by Ong et al. concluded even when comparing demographic data and outcome compared to the mono-injury cohort the only significant difference was the average inpatient stay [24].

Whilst Robinson et al. [16] did not find a significant difference in mobility scoring most studies agree on a worse ambulatory status [30] for patients with concomitant fractures also spending a longer time at rehabilitation and are less likely to be discharged home [8, 18, 27].

The difficulty is that most published studies do specify the treatment patients received for the concomitant fracture. As our treatment aim especially for our geriatric patients within our multimodular approach and geronto- trauma co-management is to enable full weight bearing and extensive range of motion we treated a high percentage of our concomitant fractures surgically and included only these in our study. To minimize anesthetics, we try to perform the second surgery within the surgery needed in any event for the hip fracture. Our mortality and complications rates seem to be comparably low. Buecking et al. [17] support our observations that functional recovery could be improved by surgical treatment of the concomitant fracture. They found the postoperative function to be restricted by the hip fracture and preexisting conditions.

Even though our sample size consisting of 85 patients with concomitant fractures is a sizable cohort there are limitations that have to be taken into account. Due to the study design as a matched pair analysis no relevant conclusions on demographic data can be drawn between the groups but the literature agrees mostly women with osteoporosis are prone to these combined injuries. As we included all concomitant fractures the cohort becomes a heterogenous group, and no valuable conclusions can be drawn to single injury patterns. Furthermore, we only evaluated the in-house mortality, and our study is lacking in long-term data.

## Conclusion

Patients with concomitant fractures must be taken into special consideration. Early surgical treatment of concomitant fractures is beneficial for patients with proximal femur fractures as it enables early mobilization and functional recovery and therefore leads to comparable mortality and complication rates even though the overall length of hospital stay is significantly increased.

## Data Availability

The data that support the findings of this study are available from the corresponding author, Annabel Fenwick, upon reasonable request.
